# The relationship of depression and quality of life with mediating role of death anxiety, silver lining and religious coping among women cancer patients in Pakistan

**DOI:** 10.3389/fonc.2025.1489169

**Published:** 2025-03-27

**Authors:** Wizra Saeed

**Affiliations:** Effat University, Jeddah, Saudi Arabia

**Keywords:** cancer patients, breast cancer, lung cancer, blood cancer depression, quality of life, death anxiety, silver lining, religious coping

## Abstract

**Objective:**

Pakistani women are among those most likely to be diagnosed with cancer. Cancer patients experience significant changes that impact their mental and physical health, primarily due to the increased burden of the disease. This study aims to explore the relationship between depression and quality of life (QOL) in cancer patients, as well as how religious coping (RC), silver lining (SL), and death anxiety (DA) influence this connection.

**Materials and methods:**

A total of 450 individuals diagnosed with cancer were recruited from outpatient departments of various hospitals in Islamabad. Out of these, 421 patients who met the inclusion criteria were included in the study. Three types of cancer were considered for data collection there was 132 (31.4%) breast cancer, 154 (36.6%) blood cancer and 135 (32.1%) lung cancer patients Participants were assessed using the following measurement tools: the Demographic Form, The Short Muslim Religious Practice and Religious Belief, the Patient Health Questionnaire (PHQ-9, 2011), the Death Anxiety Scale, the Silver Lining Questionnaire, and the WHOQOL-BREF Questionnaire.

**Results:**

The findings of the current study revealed a negative association between depression and quality of life (QOL). Additionally, death anxiety (DA) was positively correlated with both depression and QOL. Conversely, silver lining (SL) and religious coping (RC) were negatively associated with depression and positively associated with QOL. Path analysis indicated that DA, SL, and RC served as mediators in the relationship between depression and QOL among cancer patients.

**Conclusion:**

The study concluded that cancer patients can better manage their depression and enhance their quality of life by strengthening their silver lining (SL) and religious coping (RC). These findings should be considered when developing strategies to manage depression and other psychological issues in cancer patients, thereby providing more effective treatments for this population

## Introduction

Cancer is a serious illness characterized by uncontrolled cell division. Globally, it is estimated that there will be 28.4 million cancer cases by 2040 (1). Cancer is the leading cause of death worldwide, expected to account for approximately 10 million deaths in the coming years (2). In Pakistan, cancer is becoming increasingly prevalent (3).

The rapid rise in cancer diagnoses in Pakistan has become a significant public health challenge (4). According to the Global Cancer Observatory, there are 185,748 new cases, 118,631 deaths, and 390,443 cancer patients within a five-year period (5). Globally, 18.1 million new cancer cases are diagnosed each year, with 9.6 million people dying from the disease before the age of 70 (6).

Depression is one of the most frequently observed psychological symptoms in cancer patients. Research on breast cancer patients found a global depression prevalence of 32.2% (ranging from 1.8% to 89.2%) (7). Psychological issues like depression are common among cancer patients and can add an additional burden during treatment. This complicates the management and compliance with treatment. The prevalence of depressive disorders among cancer patients is two to three times higher than in the general population (8). Individuals with cancer are at a significant risk of developing depression (9).

Depression and death anxiety (DA) have a sometimes complex and biassed interaction. People who are depressed could have more death anxiety because of despair, hopelessness, or fear of the future connected with death. On the other hand, high death anxiety can aggravate depression by raising vulnerability, helplessness, and concern with death. People dealing with existential crises or life-threatening diseases, where worries about death and loss profoundly affect their emotional well-being (10).

DA, is a common psychological concern among cancer patients (11). Given the life-threatening nature of their illness and the uncertainty surrounding their prognosis, cancer patients often grapple with fears and anxieties related to death (12). DA is characterized by a vague, frightening sensation of discomfort or dread that is experienced by persons who suffer from death anxiety. This anxiety is brought on by a sense of a real or imagined threat to individual lives. While death is an inevitable part of existence for all humans, its ambiguous nature causes anxiety for cancer patients (13).

Death anxiety (DA) is a common psychological concern among cancer patients globally, but it is particularly prevalent in Pakistan due to limited treatment facilities (11). Given the life-threatening nature of their illness and the uncertainty surrounding their prognosis, cancer patients often grapple with fears and anxieties related to death (12). DA is characterized by a vague, frightening sensation of discomfort or dread experienced by individuals suffering from this anxiety, often triggered by a perceived or real threat to their lives. While death is an inevitable part of human existence, its ambiguous nature can cause heightened anxiety for cancer patients (13).

Patients with serious illnesses, such as cancer, are often confronted with the reality of death. Death anxiety may arise following a terminal diagnosis and encompasses concerns about the purpose of life, one’s legacy, and what happens after death Studies have reported several negative outcomes associated with death anxiety, including increased levels of depression and a potential decline in quality of life among cancer patients (14).

The concept of quality of life (QoL) in cancer patients is comprehensive, encompassing various dimensions such as physical, emotional, social, and psychological well-being (15, 16). Studies have shown that elevated levels of depression among cancer patients can have a detrimental effect on their QoL and ability to cope with cancer (17). The QoL of patients may also be negatively impacted by the significant side effects of numerous cancer treatments, including chemotherapy, radiation therapy, and targeted therapy (18).

Psychological conditions such as depression have a significant negative impact on quality of life (QoL) (19). Indeed, the QoL of individuals suffering from depression is markedly lower (20). Studies have shown that cancer patients with depression are at a significantly higher risk of experiencing a decline in their QoL, both overall and in specific domains (21). There is a substantial negative correlation between individuals’ overall QoL and their levels of depression (22).

Silver lining (SL) refers to the positive outcomes associated with an illness. It represents a sign of hope rather than sadness, emphasizing the ability to see a bright future despite difficult circumstances. Essentially, it involves replacing fear with optimistic thoughts (23). The silver lining effect suggests that recognizing small gains from a larger loss can improve one’s perception, making the situation feel less burdensome and enhancing quality of life and coping mechanisms for managing chronic illness (24). Quality of life (QoL) has been positively correlated with the concept of a silver lining, indicating that SL and QoL are positively related (25).

Religious coping (RC) is a technique that influences compliance with disease management and therapy in cancer patients. It involves adopting religious beliefs or practices to alleviate distress and address life’s challenges effectively (26). RC provides support when individuals face life-threatening illnesses and can help reduce stress (27). Research indicates that negative religious coping is associated with higher levels of depression and poorer emotional and physical well-being. A preliminary investigation found that psychological distress and negative religious coping were linked to a worse quality of life in cancer patients (28). In contrast, positive religious coping has been shown to improve quality of life and reduce depression and anxiety (29).Pakistan is classified as a developing country, and a significant portion of its population has been diagnosed with cancer. According to Ali et al. (4), the increasing burden of cancer is largely linked to the physical and emotional changes experienced by cancer patients. The current research aims to explore the relationship between depression (Dep) and quality of life (QoL), as well as to examine the roles of religious coping (RC), silver lining (SL), and death anxiety (DA) in mediating this relationship among cancer patients. This investigation seeks to provide a comprehensive understanding of the psychological burden faced by cancer patients (4).

The following hypotheses were formulated based on the literature review:

1. There is a negative relationship between depression (Dep) and quality of life (QoL) among cancer patients.
2. Death anxiety (DA) will act as a mediator between depression and QoL among cancer patients.
3. Silver lining (SL) will act as a mediator between depression and QoL among cancer patients.
4. Religious coping (RC) will act as a mediator between depression and QoL among cancer patients.

## Methodology

### Research design

The study was cross-sectional in nature, and data were collected from different treatment centers in Islamabad, Punjab Province, Pakistan. We used G-Power (version 3.1.9.4) to determine the sample size. The effect size was set at 0.40, the margin of error at ±0.001, and the power at 0.95, which resulted in a calculated sample size of 400 participants. Of the individuals recruited, 450 met the study’s inclusion and exclusion criteria, and ultimately, 421 participants were included in the study.

### Participants

To collect the data, purposive sampling was used. Participants with all types and stages of cancer were recruited. The sample consisted of N = 450 diagnosed female patients with cancer. Three types of the cancer were considered for data collection there was 132 (31.4%) breast cancer, 154 (36.6%) blood cancer and 135 (32.1%) lung cancer patients.

It was found that 29 of them were not eligible for inclusion because they did not fulfill the inclusion requirements or did not complete the screening procedures. Participants in the study were cancer patients who were undergoing chemotherapy regardless of stage. There was 100 (23.8%) participants of stage I, 39 (16.2%) stage II, 135 (32.1%) stage III, and 118 (100%) stage IV.

### Inclusion and exclusion criteria

The study included female cancer patients who were medically stable and capable of participating in the research. Theree types of the cancer such as Br-C (Brest Cncer), Blood Cancer (Bl-C), Lung Cancer (Lu-C) were added in the studyParticipants were recruited from all stages of the disease (Stages I–IV), ensuring that they were not in acute or critical health crises that could interfere with their ability to complete the study requirements. Inclusion was limited to individuals who were physically and cognitively able to understand and respond to survey questions or interviews. Additionally, participants were adult women within the specified age range of 18–65 years] and had provided informed consent, demonstrating their willingness to engage in the research process.

Exclusion criteria were individuals who were seriously ill, recovering from major surgery, or had severe speech impairments, physical disabilities, head injuries, or severe psychiatric comorbidities. Hospitalized patients were also excluded. Additionally, only those who showed motivation to participate and were willing to undergo psychological testing were included. Participants were required to have the ability to read, write, and communicate effectively.

### Measures

#### Demographic form

The form collected personal information from the participants, including their gender, marital status, age, education level, place of residence (owned or rented), employment status, number of earners in the household, type of cancer, age at diagnosis, patient care details, cancer stage, and treatment received.

##### The short muslim religious practice and religious belief

It is used to assess religious practices and beliefs among Muslims. Originally developed by Tayyiba AlMarri in 2009, the scale was later translated into Urdu by Ghayas and Batool (30). The SMPBS explores various religious traits and measures the extent of participation in Islamic religious activities. The scale is divided into two sections: the first section contains nine items across two subscales (religious practice and religious belief), while the second section includes five items that reflect religious conviction. For the current study, the Urdu version of the scale demonstrated a reliability coefficient of.78.

##### Patient Health Questionnaire

The Patient Health Questionnaire-9 (PHQ-9) is used to assess the severity of depression in cancer patients. This tool consists of nine items that evaluate the presence of depressive symptoms over the past two weeks, with higher scores indicating more severe symptoms. Each item is rated on a scale from 0 to 3. The PHQ-9 has demonstrated high internal consistency, with a Cronbach’s alpha of 0.89.

##### Death anxiety scale

The Death Anxiety Scale (DAS), originally developed by Templer in 1970 and later translated into Urdu by Saleem et al. (32), was used in this study to measure death anxiety in cancer patients. The scale consists of 15 items, each answered with either “true” or “false.” Scores on the DAS are interpreted as follows: a score between 9 and 15 indicates a high level of death anxiety, a score between 4 and 8 indicates a moderate level, and a score below 4 indicates no death anxiety. The scale has demonstrated reasonable internal consistency, with a Cronbach’s alpha coefficient of 0.76 and a test-retest reliability of 0.83. In the current study, the Cronbach’s alpha reliability was found to be 0.85 (31).

### Silver Lining Questionnaire

The 38-item Silver Lining Questionnaire (SLQ) is designed to assess an individual’s ability to find positive meaning in negative events and experiences (33). Originally developed to evaluate illness experiences, the scale asks participants to rate the extent to which they agree with each statement, using a scale from 1 (strongly disagree) to 5 (strongly agree). Responses of “strongly agree” and “agree” were assigned a value of 1, while all other responses were assigned a value of 0. The total score, calculated by summing these values, reflects the number of positive reflections on the situation. The SLQ demonstrated excellent reliability in the current sample, with a Cronbach’s alpha of 0.94 (34).

### WHOQOL-BREF questionnaire

The WHOQOL-BREF questionnaire was utilized to assess the quality of life (QOL) among all cancer patients. It consists of 26 items that allow for the calculation of four domain scores (35). These domain scores represent an individual’s perception of their quality of life in four areas: physical, psychological, social relationships, and environmental well-being. The scores in each domain are scaled positively, meaning that higher scores indicate a better quality of life. Prior to scoring, certain items must be reversed. To ensure comparability with the WHOQOL-100 score, the mean score is multiplied by 4. In the current study, the Cronbach’s alpha reliability for the questionnaire ranged from 0.66 to 0.84 (36).

### Procedure

Data was collected using a purposive sampling method. The study proposal was initially presented to the departmental research committee for review and certification. It was then submitted to the Bahria University Islamabad Campus Institutional Review Board (IRB) for final approval. Once authorization was obtained, data collection proceeded with the consent of the hospital administration.

In addition to providing written information on the informed consent form, the researcher also offered a brief explanation of the study’s objectives and its benefits to the participants. Participants were informed that their involvement was entirely voluntary. The hospital’s on-call oncologist directed patients to the researcher, who then recruited participants and conducted further screenings. Data were collected from Islamabad Ali Medical Center, with the entire data collection and analysis process spanning six to eight months.

### Ethical consideration

To ensure the protection of participants’ rights during data collection, the researcher kept them well-informed throughout the process. Participants reviewed the consent form thoroughly and were asked to sign it to indicate their agreement to participate in the study. The researcher also made it clear that participants could withdraw from the study at any time without any hesitation if they felt distressed. Additionally, the researcher reassured participants about the confidentiality of their information and the protection of their identities. The data collection process adhered to fundamental ethical principles, including the protection of patients’ rights and the maintenance of their dignity and respect.

### Statistical analysis

Descriptive statistics (Mean & SD) were utilized to compute the sample demographics, whilet-test were employed to compare variables. There was only one ANOVA statistic used to compare the study variables. A more thorough examination of the nature of the moderated indirect effects was conducted using the PROCESS MODMED 2.0 macro for SPSS. We use IBM SPSS Statistics Version 27.

## Results

In this study, 450 subjects were recruited from the outpatient departments of Ali Medial Center Islamabad in Punjab, Pakistan. A total of 29 individuals were removed from the study due to their failure to meet the predetermined inclusion criteria or their inability to complete the required screening procedures. Among the 421 there was 100 (23.8%) participants of stage I, 39 (16.2%) stage II, 135 (32.1%) stage III, and 118 (100%) stage IV. There was132 (31.4%) breast cancer, 154(36.6%) blood cancer and135 (32.1%)lung cancer patients. 170 (59.6%) were single and 251 (40.0%) were married.

Regarding educational qualifications, 50 (11.9%) Illiterate, 88 (20.9%) Elementary school, 78 (18.5%) Below Matric, 91 (21.6%) Matric, 64 (15.2%) Inter, and 50 (11.9%) Bachelor and above education. Participants were also categorized by their place of residence: 210 (49.9%) were from rural areas, while 211 (50.1%) were from urban areas. Duration of illness was classified as follows: 69 (16.4%) less than 6 months, 159(37.8%) one year, 147 (34.9%) two years and46 (10.9%) 3-4 years cancer patients (**Table 1**).

**Table 1:** Demographics Information.

		Frequency	Percent	Valid Percent	Cumulative Percent
Stages cancer	Stage-I	100	23.8	23.8	23.8
	Stage-II	68	16.2	16.2	39.9
	Stage-III	135	32.1	32.1	72.0
	Stage-IV	118	100.0	100.0	100
Types	Breast Cancer (Br-C)	132	31.4	31.4	31.4
	Blood Cancer (Bl-C)	154	36.6	36.6	67.9
	Lung Cancer (Lu-C)	135	32.1	32.1	100
HOI	Yes	169	40.1	40.1	40.1
	No	252	59.9	59.9	100
MS	Married	251	40.4	40.4	40.4
	Single	170	59.6	59.6	100
Edu	Illiterate	50	11.9	11.9	11.9
	Elementary	88	20.9	20.9	32.8
	Below Matric	78	18.5	18.5	51.3
	Matric	91	21.6	21.6	72.9
	Inter	64	15.2	15.2	88.1
	Bachelor and above	50	11.9	11.9	100.0
AOR	Rural	210	49.9	49.9	49.9
	Urban	211	50.1	50.1	100.0
DOI	Less than 6 months	69	16.4	16.4	16.4
	One year	159	37.8	37.8	54.2
	Two years	147	34.9	34.9	89.1
	3-4 years	46	10.9	10.9	100.0
Financial Support	Self	162	38.5	38.5	38.5
	Sibling Support	145	34.4	34.4	72.9
	Extended Family support	111	26.4	26.4	99.3
	Financial Support	3			
Available caregiver	Family member	151	35.9	35.9	35.9
	Extended family member	96	22.8	22.8	58.7
	Significant others	102	24.2	24.2	82.9
	Nursing care	72	17.1	17.1	100.0

MS, Marital Status; Edu, Education, History of Illness; DOR, Duration of Illness, AOR= Area of Residence.

There are significant mean differences in depression, death anxiety, silver lining, and religious coping for breast, lungs, and blood cancer. According to the findings of a one-way analysis of variance (ANOVA) conducted on the variables of PHQ, DA, SLS, QOL, RPSS, and RBSS across types of cancer., DA, SLS, and RPS for blood cancer, as well as the quality of life for lung cancer, were not observed to differ significantly from one another (**Table 2**).

**Table 2:** One Way Analysis of Variance Tukey Among (Types) of Cancer on Variable of PHQ, DA, SLS, QOL, RPS, and RBS among cancer patients with types of cancer(N=421).

Scales	ANOVA Statistics								Tucky test (group Comparisons			
	Types	N	Mean	SD	SE	MS	F	P	(I)	(J)	D(I-J)	P
PHQ	Br-C	132	14.50	6.83	.50	623.138	15.5048	.000	Br-C	Bl-C	± 1.758	.001
	Bl-C	154	12.74	5.38	.43	34.07			Br-C	Lu-C	± 4.29	.001
	Lu-C	135	10.20	6.31	.54				Bl-C	Lu-C	± 2.53	.006
DA	Br-C	132	10.53	2.86	.15	62.627	15.5048	.000	Br-C	Bl-C	± 758	.000
	Bl-C	154	9.77	1.21	.09	2.833			Br-C	Lu-C	± 1.37	.000
	Lu-C	135	9.17	2.02	.17				Bl-C	Lu-C	± .719	.000
SLS	Br-C	132	73.28	34.84	3.03	12389.0	15.5048	.000	Br-C	Bl-C	± 5.87	.520
	Bl-C	154	78.05	34.59	2.78	1357.86			Br-C	Lu-C	± 18.5	.000
	Lu-C	135	91.74	41.02	3.53				Bl-C	Lu-C	± 13.79	.005
QOL	Br-C	132	57.72	25.99	2.26	1042.6	15.5048	.171	Br-C	Bl-C	± 4.04	.348
	Bl-C	154	61.77	21.74	1.75	587.4			Br-C	Lu-C	± 5.34	.170
	Lu-C	135	63.07	25.13	2.16				Bl-C	Lu-C	± 1.30	.892
RPSS	Br-C	132	8.70	1.59	.13	88.48	15.5048	.000	Br-C	Bl-C	± .340	.49
	Bl-C	154	9.04	1.59	.12	4.81			Br-C	Lu-C	± 1.53	.000
	Lu-C	135	10.24	3.10	.26				Bl-C	Lu-C	± .1.19	.000
RBSS	Br-C	132	8.81	1.69	.14	217.05	15.5048	.000	Br-C	Bl-C	± .338	.683
	Bl-C	154	9.14	1.73	.13	8.30			Br-C	Lu-C	± 2.33	.000
	Lu-C	135	11.14	4.43	.38				Bl-C	Lu-C	± 2.09	.000

PHQ, Patients Health Questionnaire; QOL, Quality of Life Questionnaire; DA, Death Anxiety; SLS, Silver lining Scale; RPS, Religious Practice Subscale; RCS, Religious Belief Subscale; Br-C, (Brest Cancer); Blood Cancer, (Bl-C); Lung Cancer, (Lu-C).

Results shows that, t-test comparing single and married women on PHQ, DA, SLS, RPS, and RBS reveal that married women have higher score on SLS, QOL as compared to single women. On the other hand, single women have higher levels of PHQ, but there was no difference found on DA, QOL for married and single females. (see **Table 3**).

**Table 3:** t-test statistics between single and married women on the variable of PHQ, DA, SLS, QOL,RPS, and RBS among cancer patients with types of cancer(N=421).

	Single Women (n=170)		Married Women (n=251)		t	P	Std. Error Difference	Cohen’s *d*
	M	SD	M	SD				
PHQ-9	13.93	7.68	12.17	6.79	1.26	.000	.602	1.39
DAS	10.08	2.04	10.71	1.54	1.48	.003	.175	0.35
SLS	75.72	38.28	84.49	38.72	-2.36	.781	3.71	0.23
QOL	60.25	26.16	61.37	23.07	-.464	.000	2.41	0.05
RPSS	9.59	3.03	14.76	1.58	1.26	.000	.236	2.13
RBSS	10.20	4.29	9.33	1.68	2.91	.000	.300	0.37

PHQ, Patients Health Questionnaire; QOL, Quality of Life Questionnaire; DA, Death Anxiety; SLS, Silver lining Scale; RPS, Religious Practice Subscale; RCS, Religious Belief Subscale.

One-way ANOVA results on the variables of PHQ, DA, SLS, QOL, RPS, and RBS across different stages of cancer indicate significant mean differences in depression, death anxiety, silver lining, and religious coping for stage I,II,III, and IV for cancer patients. (**Table 4**).

**Table 4:** Descriptive statistics (M & SD) and one-way ANOVA statistics for the scale of PHQ, DAS, SLS, QOL and RC among cancer patients with Stages of cancer(N=421).

	Cancer Types	ANOVA Statistics							Tucky test (Group Comparisons)			
		N	Mean	(SD)	(SE)	MS	F	P	(I)	(J)	D(I-J)	P
DAS	Stage-I	94	8.91	1.70	.17	37.15	12.92	.000	Stg-I	Stg-II	± 1.04	.000
	Stage-II	115	9.95	1.33	.12	2.87			Stg-I	Stg-III	± 1.45	.000
	Stage-III	100	10.37	2.01	.20				Stg-I	Stg-IV	± 1.04	.000
	Stage-IV	112	9.95	1.70	.16				Stg-II	Stg-III	± .41	.282
	–	–	–	–					Stg-II	Stg-IV	± .00	1.00
	–	–	–	–					Stg-III	Stg-IV	± .41	.280
PHQ-9	Stage-I	94	9.28	5.37	.55	524.08	15.70	.000	Stg-I	Stg-II	± 3.39	.000
	Stage-II	115	12.63	5.36	.49	33.37			Stg-I	Stg-III	± 5.64	.000
	Stage-III	100	14.93	6.44	.64				Stg-I	Stg-IV	± 3.4	.000
	Stage-IV	112	12.76	5.88	.55				Stg-II	Stg-III	± 2.25	.024
									Stg-II	Stg-IV	± .089	.119
									Stg-III	Stg-IV	± 2.16	.034
SLS	Stage-I	94	88.44	41.14	4.24	5119.41	3.70	.012	Stg-I	Stg-II	2.77724	.950
	Stage-II	115	85.66	37.08	3.45	1383.71			Stg-I	Stg-III	14.36681^*^	.037
	Stage-III	100	74.08	35.76	3.57				Stg-I	Stg-IV	12.49145	.079
	Stage-IV	112	75.95	35.02	3.30				Stg-II	Stg-III	± 11.58957	.105
									Stg-II	Stg-IV	± 9.71421	.202
									Stg-III	Stg-IV	± 1.87536	.983
QOL	Stage-I	94	71.11	20.99	2.16	6136.1	11.16	.000	Stg-I	Stg-II	± 9.70	.016
	Stage-II	115	61.41	22.75	2.12	549.75			Stg-I	Stg-III	± 19.43	.000
	Stage-III	100	51.67	24.56	2.45				Stg-I	Stg-IV	± 11.00	.005
	Stage-IV	112	60.10	25.02	2.36				Stg-II	Stg-III	± 9.73	.014
									Stg-II	Stg-IV	± 1.30	.975
									Stg-III	Stg-IV	± 8.42	.046
RPSS	Stage-I	94	10.58	2.89	.29	197.790	24.891	.000	Stg-I	Stg-II	± 1.59	.000
	Stage-II	115	8.99	1.39	.13	7.946			Stg-I	Stg-III	± 2.02	.000
	Stage-III	100	8.56	1.80	.18				Stg-I	Stg-IV	± 1.29	.000
	Stage-IV	112	9.28	2.40	.22				Stg-II	Stg-III	± .43	.468
									Stg-II	Stg-IV	± .29	.737
									Stg-III	Stg-IV	± .72	.073
RBSS	Stage-I	94	11.81	4.73	.48	542.138	23.212	.000	Stg-I	Stg-II	± 2.75	.000
	Stage-II	115	9.09	1.50	.14	23.356			Stg-I	Stg-III	± 3.17	.000
	Stage-III	100	8.68	1.97	.19				Stg-I	Stg-IV	± 2.48	.000
	Stage-IV	112	9.36	2.28	.21				Stg-II	Stg-III	± .41	.703
									Stg-II	Stg-IV	± .27	.888
									Stg-III	Stg-IV	± .68	.290
REL	Stage-I	94	22.55	7.44	.76	37.15	12.92	.000	Stg-I	Stg-II	± 4.57	.000
	Stage-II	115	17.98	2.56	.23	2.87			Stg-I	Stg-III	± 5.31	.000
	Stage-III	100	17.24	3.76	.37				Stg-I	Stg-IV	± 3.86^*^	.000
	Stage-IV	112	18.68	4.67	.44				Stg-II	Stg-III	± .74	.66
									Stg-II	Stg-IV	± .704	.69
									Stg-III	Stg-IV	± 1.45	.13

PHQ, Patients Health Questionnaire; QOL, Quality of Life Questionnaire; DA, Death Anxiety; SLS, Silver lining Scale; RPS, Religious Practice Subscale; RCS, Religious Belief Subscale; REL, Religiosity.

According to the findings, there is a significant inverse relationship between PHQ and QOL, which means that an increase in PHQ will result in a decrease in QOL and vice versa. Depression, on the other hand, has a considerable positive connection with both DA and R. According to the findings, there was a clear and significant negative relationship between PHQ and SLS. In addition, the findings indicate that DA, SLS, and RC have a role in mediating the connection between PHQ and QOL. (**Table 5**).

**Table 5:** QOL, RCS and REL among Cancer Patients (N=421).

Predictors	DA			SLS			REG			QOL		
	*Coeff.*	*SE*	*P*	*Coeff.*	*Coeff.*	*SE*	*P*	*SE*	*P*	*Coeff.*	*SE*	*P*
Constant	*6.688*	.*099*	*67.27*	*134.75*	*25.69*	*.453*	*56.71*	*3.00*	*44.83*	*197.37*	*8.40*	*23.49*
PHQ	*.251*	*.007*	*35.04*	-4.311	*-.535*	*.032*	*16.40*	.216	-19.90	-2.36	.276	-8.54
DA										-7.20	.834	-8.63
SLS										*-.216*	*.027*	*-7.78*
RCS										*-.9874*	*.184*	*-.5.36*
	*R^2^* =.*8635*			*R^2^* = *.6971*			*R^2^* = .924			*R^2^*_=_ *.782*		
	*F*(1,419)= *1227.8*, *p*<.001			*F*(1,419) =*396.2*, *p*<.001			*F*(1,419)= 654.95, *p*<.001			*F*(4, 416)= 164.15, *p*<.001		

PHQ, Patients Health Questionnaire; QOL, Quality of Life Questionnaire; DA, Death Anxiety; SLS, Silver lining Scale; RCS, Religious Coping Scale.

The results from the indirect effect reveal that DA, SLS and RC were found to be significant mediators between PHQ and QOL. Further, this reflects that the increase in DA will leads to increase in PHQ and decrease in QOL. On the other hand, increase in SLS and RC will decrease. (**Table 6**).

**Table 6:** Indirect effects of PHQ, DA, SLS, QOL, and RCS among Patients of Cancer (N=421).

Mediator	Effect	BootsSE	95%BootCI	
			*BootLL*	*Boot*
	-.348	.312	-.921	.307
DA	-1.808	.271	-2.292	-1.236
SLS	*.931*	*.145*	*.665*	*1.231*
RCS	*.529*	*.1355*	*.268*	*.799*

Effect, standardized regression coefficient; BootCI, bootstrapped confidence interval; BootLL, bootstrapped lower limit; BootUL, bootstrapped upper limit; DA, Death Anxiety; SLS, Silver lining Scale, RCS, Religious Coping Scale.

**Figure 1:**
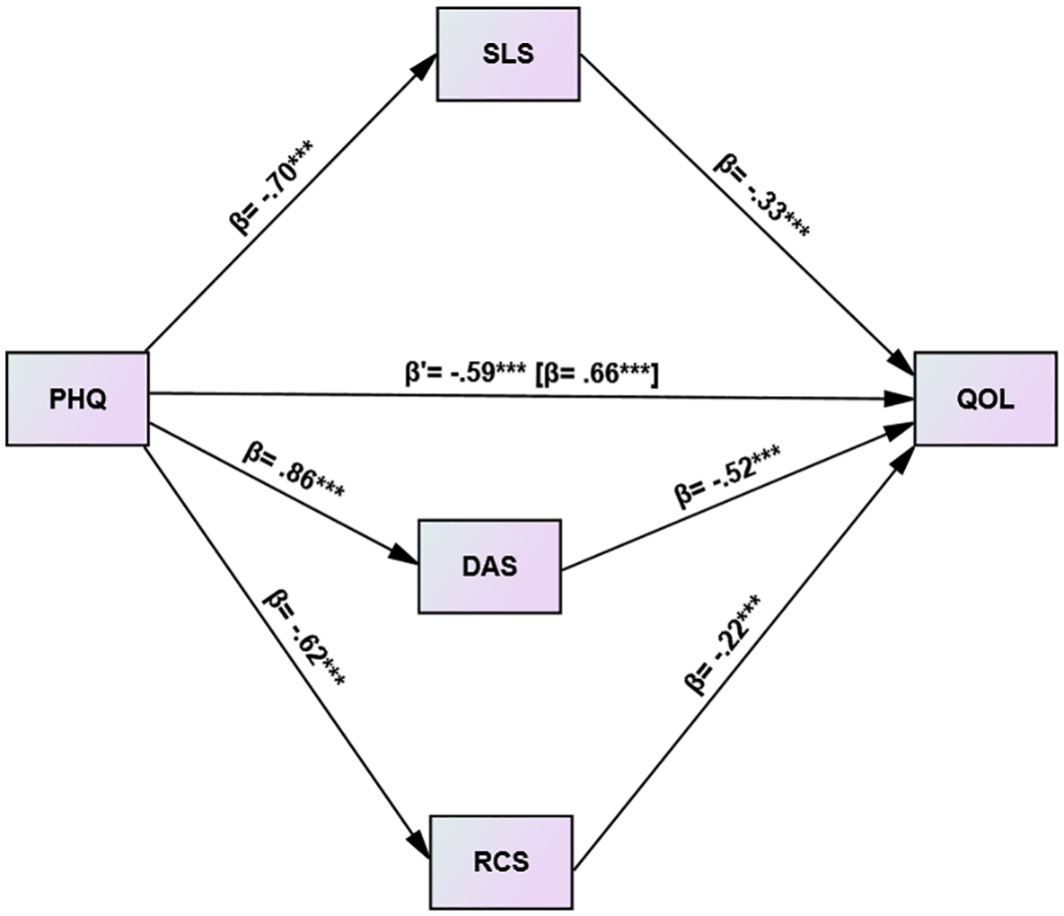
Depression and QOL among cancer patients: the mediating role of DA, SLS and RC (N= 421). ***p<0.001.

The study examines the mediating effect of three variables, namely, DA, SL, and RC in the relationship between depression and QOL among cancer patients. The reported coefficients are standardized (β). The paths in the model represent the connections between depression and the mediators. The b paths show the relationships between the mediators and the quality of life (QOL). The c path represents the association between depression and QOL, while the c’ path indicates the relationship between depression and QOL when the mediators are considered (**Figure 1**).

## Discussion

This study enhances the understanding of the relationship between depression and quality of life (QOL) among cancer patients and examines the mediating roles of religious coping (RC), death anxiety (DA), and silver lining (SL). Our results indicate that depression negatively impacts QOL in cancer patients, with higher depression scores correlating with decreased QOL. Research shows that depression significantly affects cancer patients’ quality of life, complicating daily activities and coping with illness. Depression is associated with a reduced quality of life and a lower chance of survival (37). As a clinical condition, depression can extend hospital stays, complicate care management, and further diminish QOL (38). QOL in cancer patients is linked to their perceptions of physical, emotional, social, and mental functioning, as well as their overall life satisfaction throughout the disease course. Cancer patients generally experience a lower quality of life compared to the general population (39).

We examined the mediating role of death anxiety (DA) in the relationship between depression and quality of life (QOL). Our results indicate a positive relationship between depression and DA, meaning that increased depression is associated with heightened DA, while decreased depression corresponds with lower DA. Research supports this correlation between depression and death anxiety (8), as unresolved suffering—both physical and emotional—can exacerbate these feelings (40). Additionally, we found a negative correlation between DA and QOL: as DA increases, QOL decreases, and vice versa. A cancer diagnosis often leads to significant changes in a person’s physical, mental, and social well-being, which can heighten death anxiety during challenging times. DA is increasingly recognized as a major factor affecting mental health and the ability to adapt to life changes, thereby reducing QOL among cancer patients (41). Our study also demonstrated that DA mediates the relationship between depression and QOL, with DA’s role significantly and negatively impacting both depression and QOL.

Results found that silver lining (SL) mediates the relationship between depression and quality of life (QOL) in cancer patients. Our results revealed a negative correlation between depression and SL, indicating that as depression increases, the perception of finding positive aspects (SL) decreases. Depression reduce the cognitive flexibility and positive thinking which impairs the ability to find silver lining in life challenges such as suffering from cancer (43). On the other hand, actively seeking and focusing on positive aspects of cancer can lead to greater value and satisfaction in daily activities (44), as well as more positive health-related behaviors among cancer patients (42). Additionally, our findings show a significant positive correlation between SL and QOL: higher levels of SL are associated with improved QOL among cancer patients (24). SL fosters positive feelings such as optimism, hope, coping strategies, and social support, which subsequently reduce emotional discomfort and loneliness, hence improving the quality of life for cancer patients. (45),. This underscores the importance of SL in enhancing the well-being of cancer patients by mitigating the impact of depression and improving their overall quality of life.

Current study indicates that religious coping (RC) acts as a mediator between depression and quality of life (QOL) among cancer patients. We found that depression and religious coping has bidirectional relationship. individuals who engage more in religious coping strategies are less likely to experience severe depressive symptoms and vice versa (46). Individuals with depression demonstrate a lack of enthusiasm for religious coping, a sense of pessimism, and a reduced energy level, thereby challenging their faith and religious beliefs. (47). Depressive feelings and anxiety are exacerbated by a reduced sense of purpose, which is a result of lower religious coping.

(48). Additionally, RC positively correlates with QOL; individuals with stronger religious coping abilities tend to have a better quality of life compared to those with weaker coping abilities (49 Participation in religious activities fosters a sense of purpose, optimism, positive emotions, and social support. These techniques facilitate stress reduction, enhance self-esteem, and foster hope, so improving the quality of life among cancer patients.

(50, 51). Our study further reveals that RC significantly and positively mediates the relationship between depression and QOL. Religious coping enhances mental health and enables individuals to confront challenges in their lives with more resilience and confidence, ultimately resulting in a more rewarding, meaningful life and higher quality of life.

Furthermore, study compared single and married women in terms of their scores on the Patient Health Questionnaire-9 (PHQ), Death Anxiety Scale (DAS), Silver Lining Scale (SLS), Religious Practice Scale (RPS), and Religious Belief Scale (RBS). The results reveal that married women scored higher on the SLS and QOL compared to single women. Married women are more likely to achieve high scores on QOL and SL as a result of financial stability, emotional support, and fulfillment. They have fostered a more robust sense of direction and shared responsibility, which has contributed to an overall improvement in QOL. (52, 53). Conversely, single women exhibited higher levels of depression (PHQ). However, no significant differences were found between single and married women in terms of Death Anxiety (DA) and Single women are at a higher risk of depression as a result of financial distress, increased burden, and emotional isolation. (54.), It has further analyzed the mean differences in PHQ, DA, SLS, QOL, RPS, and RBS across different stages of cancer (Stage I, II, III, and IV). The results indicate significant mean differences in depression, death anxiety, silver lining, and religious coping among cancer patients at various stages of the disease.

## Conclusion

The findings of this study highlight the broad spectrum of psychological challenges faced by cancer patients, particularly depression, which significantly undermines their quality of life. Our investigation into the role of death anxiety, silver lining, and religious coping strategies in Pakistan reveals that depression has a detrimental effect on the quality of life for cancer patients. DA negatively impacts both depression and quality of life, exacerbating the psychological burden on cancer patients.SL and RC both positively influence depression and quality of life. SL and RC act as mediators, improving quality of life and mitigating the adverse effects of depression. Our study concludes that enhancing silver lining and religious coping strategies can help cancer patients better manage depression and improve their overall quality of life. These insights should be considered in developing comprehensive strategies for addressing depression and other psychological issues in cancer patients, potentially leading to more effective interventions and support.

## Strengths and limitations of the study

This study possesses several strengths, including its distinctive emphasis on the intricate interactions among depression, quality of life, social support, resilience, and distress in cancer patients. A thorough theoretical framework that integrates DA, RC, and SL perspectives enhances the analysis. The varied sample of cancer patients improves generalizability and offers a comprehensive picture of individuals with cancer. Apart from the strength of the study current research is limited by several constraints that must be considered. Firstly, the study focused exclusively on female cancer patients, and participants were recruited solely from outpatient departments. The research specifically examined quality of life, depression, death anxiety, finding the silver lining, and religious coping strategies. Several variables were not evaluated, including patients’ coping mechanisms, psychological and social support systems, concurrent medical problems, confirmed psychiatric diseases, and the impact of aging. Additionally, no interventions were provided to address these psychological issues. Another limitation was the inability to enroll enough participants due to the restricted amount of time available for the study.

## Recommendation and implication of the study

The sample size was limited for this study, thus future researchers may perform research on a larger sample to generalize the findings of the study. It was a correlation study where we find relationship between variables but interventional research on this topic should take into consideration specific cognitive and behavioral exercises as well as support from religious practices as the main strategies to assist patients in improving their QOL and reducing their levels of depression in order to better cope with this lethal illness by having strong religious coping and silverling This study provides mental health professionals with critically important background, when it comes to the management of patients suffering from cancer and additional persistent medical conditions, such as diabetes, stroke, etc.

## Data Availability

The original contributions presented in the study are included in the article/supplementary material. Further inquiries can be directed to the corresponding author.
